# Palliative care in day-hospital for advanced cancer patients: a study protocol for a multicentre randomized controlled trial

**DOI:** 10.1186/s12904-021-00754-x

**Published:** 2021-04-17

**Authors:** Laura Marret, Amélie Anota, Lorraine Waechter, Celine Laouisset, Timothee Marchal, Alexis Burnod, Elisabeth Angellier, Oum El Kheir Djoumakh, Clemence Thebaut, Anne Brédart, Sylvie Dolbeault, Jean-Christophe Mino, Carole Bouleuc

**Affiliations:** 1 Department of Supportive and Palliative Care, Institut Curie, Paris, France; 2 Biostatistics Unit, DRCI, Centre Léon Bérard, Lyon, France; 3 French National Platform Quality of Life and Cancer, Lyon, France; 4 INSERM, EFS-BFC, UMR 1098- Université de Bourgogne-Franche-Comté, Besançon, France; 5 Department of Supportive and Palliative Care, Hôpital Cochin, Paris, France; 6 Department of Supportive and Palliative Care, Institut Curie, Saint Cloud, Paris, France; 7 Methodological and Quality of Life Unit in Oncology (INSERM 1098), University Hospital, Besançon, France; 8 Université de Limoges, UMR 1094 (NET), Limoges, France; 9 Université Paris-Dauphine, PSL Research, University, LEDa [Legos], Paris, France; 10 Department of Psycho-Oncology, Institut Curie, Paris, France; 11 Laboratoire Psychopathologie et Processus de Santé, Université de Paris, F-92100, Boulogne Billancourt, Paris, France; 12 Department of Medical Ethics, Sorbonne University Medical School, Paris, France

**Keywords:** Quality of life, Palliative care, End-of-life care, Advanced cancer, Study design

## Abstract

**Background:**

Team-based and timely integrated palliative care is a gold standard of care in oncology, but issues concerning its optimal organization remain. Palliative Care in Day-Hospital (PCDH) could be one of the most efficient service model of palliative care to deliver interdisciplinary and multidimensional care addressing the complex supportive care needs of patients with advanced cancer. We hypothesize that, compared to conventional outpatient palliative care, PCDH allows the clinical benefits of palliative care to be enhanced.

**Methods/design:**

This study is a multicentre parallel group trial with stratified randomization. Patient management in PCDH will be compared to conventional outpatient palliative care. The inclusion criteria are advanced cancer patients referred to a palliative care team with an estimated life expectancy of more than 2 months and less than 1 year. The primary endpoint is health-related quality of life with deterioration-free survival based on the EORTC QLQ-C30 questionnaire. The secondary objectives are the following: increase in patient satisfaction with care using the EORTC PATSAT-C33 and OUT-PATSAT7 questionnaires, better understanding of the prognosis using the PTPQ questionnaire and advance care planning; decrease in the need for supportive care among relatives using the SCNS-P&C-F questionnaire, and reduction in end-of-life care aggressiveness. Patients will complete one to five questionnaires on a tablet before each monthly visit over 6 months and will be followed for 1 year. A qualitative study will take place, aiming to understand the specificity of palliative care management in PCDH. Cost-effectiveness, cost-utility and, an additional economic evaluation based on capability approach will be conducted from a societal point of view***.***

**Discussion:**

The first strength of this study is that it combines the main relevant outcomes assessing integrated palliative care; patient quality of life and satisfaction; discussion of the prognosis and advance care planning, family well-being and end-of-life care aggressiveness. The second strength of the study is that it is a mixed-method study associating a qualitative analysis of the specificity of PCDH organization, with a medical-economic study to analyse the cost of care.

**Trial registration:**

**Name of the registry:** IDRCB 2019-A03116–51

**Trial registration number:** NCT04604873

**Date of registration:** October 27, 2020

**URL of trial registry record**

## Background

Evidence on the efficacy of an early integration of palliative care (PC) has emerged in recent years for patients with advanced cancer. A recent Cochrane meta-analysis identified seven eligible randomised clinical trials comparing the effects of early PC interventions with standard cancer care [1]. This review showed significant beneficial effects on Health-Related Quality of Life (HRQoL) and on symptom intensity among patients, but the effects on depression and mortality remained uncertain. Following this meta-analysis, three studies were published confirming the clinical benefit of early palliative management [2–4] whilst a Danish study reached negative conclusions [5]. Improvement in various outcomes reported by families, such as HRQoL, perception of burden, psychological distress or social well-being, has been also demonstrated in randomised studies [6–10]. Considering this robust scientific evidence, the American Society of Clinical Oncology (ASCO) first published a provisional opinion, which then became official guidelines, validating early interventions carried out by multidisciplinary PC teams for patients with advanced cancer [11, 12]. PC interventions were also found to reduce end-of-life (EOL) care aggressiveness on the basis of validated criteria. These indicators are the occurrence in the last 30 days before death of systemic anti-cancer treatments, emergency visits or hospitalizations, intensive care unit admissions, PC unit admissions and the length of stay [13–16]. Regarding these criteria, several non-randomized studies have also demonstrated the impact of PC on EOL care aggressiveness [17–20]. Only 3 randomized clinical trials comparing early PC with standard of care have conducted assessments of EOL care aggressiveness; two of them found significant results, mainly more extensive use of hospice care or lower incidence of chemotherapy in the last 14 days before death [21–23]. This reduction in EOL care intensity could also have medical and economic consequences. In a Canadian study, PC interventions reduced EOL healthcare costs by limiting the occurrence and the length of stay in hospital and intensive care unit, specific anti-cancer treatments that have become ineffective and medications that have become non-essential [24–27]. However, exact conditions for integrated PC need to be clarified. Consensus had been found for statement of essential components of PC: rapport and relationship-building with patient and family caregivers, symptom management, exploration of understanding and education about the illness and prognosis and clarification of treatment goals, assessment of and support for coping, assistance with medical decision-making and coordination with other care providers [12–28]. The optimal timing of specialist palliative care referral remains unclear but likely depends on the individual patient and the health care system [29]. The ASCO guidelines suggest that early PC instatement should ideally start within 8 weeks after the diagnosis of advanced cancer defined as patients with a life expectancy between 6 and 24 months [12]. Given the limited PC staff and structures, this recommendation is infeasible, so that the concept of early PC has shifted towards timely and targeted palliative care integration. Thus, instead of early palliative care for all, experts advocate for timely palliative care, selecting the right patient for the right level of intervention at the right time. The optimal model may be the use of standardized need-based criteria to trigger a referral for patients who are most appropriate for specialist palliative care in the outpatient setting [29, 30]. Ultimately, efficient care model for palliative care delivered in the outpatient setting is not yet well-defined [30, 31]. There is heterogeneity in trial design among nature and setting of palliative care interventions, with some studies involving interdisciplinary palliative care teams and others involving nurse-led palliative [28]. Some teams also include a social worker, a chaplain, and/or a rehabilitation specialist [12, 27]. However, to date, none study has directly compared interdisciplinary teams with single-practitioner–led models, and further research is needed.

Palliative care in day-hospital (PCDH) settings could enhance the efficacy of integrated PC interventions compared to standard outpatient PC consultations [32]. PCDH should be distinguished from the palliative day-care unit as “a model [ …] which enables patients to receive physiotherapy and occupational / music / art therapy; to meet with others in similar situations to themselves in a friendly social /non-clinical environment” [33]. PCDH assessed in this study is a medical unit in charge of symptom assessment and relief, as well as delivery of information and shared decision-making, promoting home stay for patients with advanced cancer who so wish. The advantages of PCDH in PC delivery are numerous; its management by both a PC physician and a nurse increases support and coaching possibilities for patients and their relatives, and allows immediate medical exploration and symptom management; interviews including an oncologist enable consultations prior to shared medical decisions on oncological treatments; a longer length of stay favours discussion of prognosis, EOL management and advance care planning; the presence of nurses favours coordination with supportive care professionals and liaising with home health-care professionals. The aim of this study is to demonstrate that PCDH is a new mode of PC delivery that could enhance the efficacy of integrated PC for advanced cancer patients. A randomized controlled trial will be conducted comparing PCDH with PC outpatient consultations, and assessing patient HRQoL and patient-related outcomes, EOL care aggressiveness and its medical and economic impact. A mixed-method is to be used, adding a qualitative study, aiming to better understand factors contributing to satisfaction with care in patients depending on the model of PC received, the PCDH organization and to examine the hypothesised enhancement of PC interventions with the PCDH model.

## Methods

### Setting

The study will be conducted at four Comprehensive Cancer Centres in France (Institut Curie in Paris and Saint-Cloud, Centre Lacassagne in Nice, Institut Bergonié in Bordeaux, and Centre Paul Strauss in Strasbourg). The medical turnover of the different centres is variable, ranging from 1000 to 5000 new patients per year, with breast cancer accounting for around 50% of the primary tumours. Each centre has a PC team integrated into overall cancer care. Referral to the PC team is triggered by oncologists for advanced cancer patients, according to usual criteria, such as severe physical symptoms and/or psychosocial distress. PC delivery follows international guidelines and current best practices.

### Trial design

This study is a mixed-method phase-3 randomized trial comparing two different organisations of PC delivery for patients with advanced cancer. According to the Medical Research Council framework, mixed-methods are recommended for complex interventions [34]. The quantitative study is a multi-centre parallel-group open-label randomised trial, comparing PCDH (experimental group) to standard outpatient PC (control group). The concurrent nested qualitative study includes semi-structured interviews with patients and caregivers. It will probe the perceptions of patients, family members and PC teams involved in PCDH. A medical and economic study will evaluate the cost-effectiveness and the cost-utility ratios of the PCDH system compared with standard outpatient PC.

**Fig. 1:**
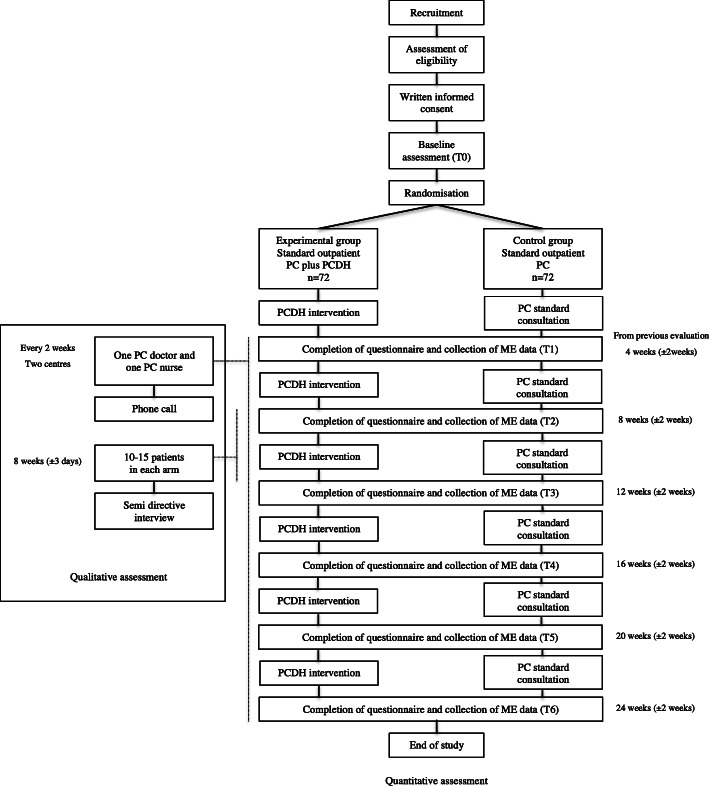
Flowchart. Abbreviations. ME: Medical-Economic; PC: Palliative Care; PCDH: Palliative Care Day-Hospital

The flow chart for the Randomized Controlled Trial (RCT) is shown in Fig. 1. The study protocol and other required documents were reviewed and approved by the medical ethics committee, Paris (date: 2 March 2020, number: 2019-A03116–5).

### Intervention: palliative Care in day Hospital

In the course of a 2 to 4-hour stay in PCDH, patients and their families are cared for by the PC team composed of a doctor and a nurse, with a standardized procedure:

- Assessment of palliative care needs, carried out by the palliative care team (30 to 60 min)
- Intervention of at least 2 supportive care professionals who can be psychologists or psychiatrists, social workers, physiotherapists, or dieticians (30 to 60 min)
- Intervention by an oncologist if needed, often in a joint meeting with the PC team (15 to 30 min)
- Complementary investigations if needed (biology, radiology, medical imaging)
- Technical care if needed (central venous catheters installation, transcutaneous electrical neuro-stimulation sessions, complex dressing of malignant wounds, pleural or ascites evacuating puncture.
- A specific time allocated to focus on patients’ needs for information, such as shared decision-making, the aims of anti-cancer treatment, the risk of complications, prognosis, and advance care planning (30 to 60 min)
- A specific time allocated for the PC team to liaise with the home health-care professionals (30 to 60 min)

The main characteristics of PC delivery in PCDH are summarised in Table 1.

**Table 1:** Comparison of the conditions of care delivery between standard outpatient PC and PCDH

	Standard outpatient PC	PCDH
Symptom relief	- Advice and medication - Planning for additional tests and specialized consultations	- Advice and Medication - Planning for additional tests and specialized consultations - Immediate titration or imaging if needed - Supportive care team intervention - Pleural or ascites evacuation
Relationship building and education	Consultation length < 30–45 min with the PC physician	- Consultation duration > 2 h with the PC team, physician and nurse - Dedicated time for relatives - Dedicated time for care coordination
Consultation and decision-making	PC physician for a short time	- Oncologist and PC physician - Longer time for discussion
Advance care planning	PC physician for a short time	- PC physician and nurse - Longer time for discussion

Abbreviations. *ME* Medical-Economic; *PC* Palliative Care; *PCDH* Palliative Care Day-Hospital

### Procedure

Patients are invited to enrol at the time of their first consultation with the palliative care team and are asked to complete questionnaires (baseline data) after providing written informed consent. Patients are also invited to designate a family member or person close who can assist them during palliative care visits and who will also complete questionnaires.

Patients randomly allocated to the experimental group or control group are seen in protocol visit monthly. For patients in experimental group with no indication for PCDH admission (intervention needed by at least 2 supportive healthcare professionals, or patients with severe distress), an outpatient PC consultation is planned. Patients randomized in the control group attend monthly outpatient consultations. Supportive care interventions, involving a psychologist, a psychiatrist, a social worker, a dietician, or a physiotherapist are scheduled at any time as needed. In both groups, patients can undergo emergency or scheduled hospitalization. The duration of the study is 6 months, and the duration of follow up is 6 months.

### Eligibility criteria

The inclusion criteria are as follows: being over 18 years old with an advanced solid tumour, a performance status of 2 on the East Cooperative Oncology Group (ECOG) scale, addressed for the first time to the palliative care team, having an estimated life expectancy of more than 2 months and fewer than 12 months; able to communicate in French and answer questionnaires, affiliated to a social security system, and having signed informed consent. The exclusion criteria concern patients with primitive brain tumour or malignant hemopathy, or severe psychopathological disorders, inability to carry out follow-up even by phone at the cancer center until death, person deprived from liberty, pregnant patient or childbearing potential without effective contraception.

### Objectives

The main objective is to show that, compared to standard integrated PC, PCDH sessions can enhance the positive impact of integrated PC on patient HRQoL. The secondary objectives are to show that, compared to standard integrated PC, PC in day hospital can:

- Alleviate caregiver burden
- Decrease anxious and depressive symptoms
- Improve satisfaction with care
- Improve patients’ awareness of their prognosis and encourage advanced care planning
- Decrease the aggressiveness of EOL care
- Decrease the cost of care in the 3 months before death

### Endpoints

The primary outcome is deterioration-free survival (QFS) in PCDH with a three dimensions of the European Organization for Research and Treatment of Cancer Quality of Life Questionnaire Core30 (EORTC QLQ-C30) questionnaire targeted as co-primary endpoints: global health status, fatigue, and emotional functioning.

Secondary endpoints are the following:

- Aggressiveness of EOL care according to usual criteria: in the last months before death, emergency consultations or hospitalizations, intensive care unit admissions, levels of anticancer treatment, place of death and length of stay in PC unit,
- Satisfaction with care according to the EORTC Out-Patient Satisfaction Core 33questionnaire (PATSAT-C33) and Out-patient satisfaction 7 (OUT-PATSAT7) questionnaires,
- Depressive and anxiety symptoms according to the Hospital Anxiety Depression Scale questionnaire (HADS),
- Patient awareness of negative prognosis according to the Prognosis and Treatment Perception Questionnaire (PTPQ),
- Advanced care planning,
- Needs for supportive care on the part of relatives according to the Supportive Care Needs Survey for Partners and Caregivers questionnaire (SCNS32-P&C-F),
- Overall survival,
- Direct costs of care (hospitalizations, consultations, home hospitalization, networks, anti-cancer treatments).

### Patient-related outcomes

Patients and their optionally designated relative complete the questionnaires before each monthly visit according to a schedule described in Table 2.

**Table 2:** Schedule of events

Assessments	Screening (V1)	6-month period						Follow-up period
Study Day visit window in days)	Day 1	V2	V3	V4	V5	V6	V7	
Informed consent	X	**4 weeks** **±2 weeks**	**8 weeks** **±2 weeks**	**12 weeks** **±2 weeks**	**16 weeks** **±2 weeks**	**20 weeks** **±2 weeks**	**24 weeks** **±2 weeks**	24-**104 weeks** **±2 weeks**
Inclusion/Exclusion criteria	X							
Randomization	X							
**Patient**								
EORTC QLQ- C30	X	X	X	X	X	X	X	
PATSAT-C33 OUTPATSAT7					X		X	
PTPQ	X			X			X	
HADS	X			X			X	
ICECAP-SCM	X		X		X		X	
Medical-economic data assessment	X	X	X	X	X	X	X	X
End-of-life data collectionª		X	X	X	X	X	X	X
**Primary caregiver**								
SCNS-P&C	X		X		X		X	

a if applicable

- The EORTC QLQ-C30 is a validated HRQoL questionnaire for cancer patients. This questionnaire generates fifteen HRQoL scores: one global health status score, five functional scores (physical, role, emotional, cognitive and social) and nine symptom scores (fatigue, nausea and vomiting, pain, dyspnea, insomnia, loss of appetite, constipation, diarrhoea and financial difficulties) [35]. Each score is standardized on a 0 to 100 scale, and a high of global health status, a high functional level, and a high symptomatic level.

- The PATSAT-C33 is a questionnaire on satisfaction with care, completed by a specific OUT-PATSAT7 module for ambulatory care [36, 37]. Both the questionnaire and module measure cancer patient satisfaction with the care provided by doctors and nurses, and their satisfaction with care organization and services.

-The HADS questionnaire is composed of 14 items, including 7 items related to anxiety and 7 to depression [38]. A score is generated for each dimension. These scores range from 0 to 21. A score from 0 to 7 corresponds to a normal level of anxiety-depression, 8 to 10 a moderate level of anxiety-depression and 15 to 21 a severe level of anxiety and depression.

- The PTPQ consists of 13 items evaluating patients’ information preferences, perceptions of prognosis, the aims of cancer treatments, and communication about end-of-life care [39]. The PTPQ evaluates patients’ beliefs regarding: 1) the probability of a cure, 2) the importance and usefulness of knowing the prognosis, 3) the main objective of cancer care, 4) preference for treatment information, and 5) satisfaction with the quality of information received on prognosis and treatment.

-The SCNS32-P&C-F questionnaire assesses the impact of care on the unmet needs of family members and caregivers who are the main source of emotional, physical and social support for patients on a daily basis [40, 41]. This questionnaire assesses the needs for information on care for their own health, their emotional and psychological needs, social needs, the consequences of illness at work, and communication and support needs. Each item is assessed using a score ranging from 1-no need for support to 4 unmet needs for support.

-The Index of Capability Supportive Care Measure Scale (ICECAP-SCM) provides a measure of well-being at the end of life [42–44]. It is specifically designed for use in a palliative care setting. It was developed in the United Kingdom using extensive qualitative research. This questionnaire comprises 7 items: 1/ ability to express oneself about one’s care, 2/ family environment, 3/ physical suffering, 4/ psychological suffering, 5/ individual dignity, 6/ help and support, and 7/ preparing for death, each with 5 possible answers.

### Randomisation

Patients are randomized between the PCDH group (experimental group) and standard outpatient PC group (control group). Assignment is determined by the random generation of a 1:1 randomization sequence. Randomization is performed by minimization, with stratification according to the following criteria:

- The cancer centre, as unknown heterogeneity in PC management could induce a bias.

- The presence or absence of a designated person close, to obtain a well-balanced population in the two groups, enabling valid statistical analyses for the caregiver-related outcomes.

- The main indicators influencing prognosis and HRQoL: age > 75 or 75 and over, primary cancer, of the breast /prostate type and life expectancy > or < 6 months.

### Statistical analysis

Assuming an improvement in the median time of non-deteriorating quality of life from 1 month to 2 months; with a bilateral Type I error of 0.0166 and a statistical power of 80%; for a ratio of 1: 1 (H0: HR = 1 / H1: HR = 0.5), the number of patients required is 96 to observe 88 events. With an attrition rate of 50% (patients lost to follow-up or prematurely deceased), the number of patients required is 144, meaning 72 patients in each arm.

The superiority of one type of care over the other will need to be demonstrated if at least one of the three HRQoL dimensions targeted show a significantly longer deterioration free survival compared to the other group without significantly shorter deterioration on at least one of the other two HRQoL dimensions [45, 46]. The scores for each dimension will be analysed separately. Quality of life survival deterioration free survival (QFS) will be defined as the time interval between inclusion in the study and the occurrence of the first clinically significant deterioration of at least 10 points from the baseline score, without further improvement of at least 10 points from the baseline score, or death [47].

The analysis of the primary endpoint will be performed at the statistical level α = 0.0166 and the analysis of the secondary endpoints will be performed at the level α = 0.05. The minimal clinically important difference will be defined as 10 points for each dimension of the QLQ-C30.

Analyses will be conducted in intent-to-treat and will include all randomized patients, and analysed according to the group they were assigned, regardless of what intervention they really received and the respect of not of eligibility criteria.

A secondary analysis will be carried out per protocol after exclusion of individuals presenting major deviations from the protocol and include patients who respect the intervention group allocated. The description of the patients for all socio-demographic and clinical characteristics, as well as the scores obtained on the different questionnaires will be based for the two intervention groups on the data collected at inclusion. The categorical variables will be expressed as absolute and relative frequencies and the continuous variables as means (standard deviations) or medians (min/max).

QFS will be estimated according to the Kaplan-Meier method and described per group using the median and confidence interval at (100-α) %. The comparison between the two RCT groups will be performed using log-rank test. A univariate Cox model will be performed to estimate the Hazard Ratio coefficient and its confidence interval at (100-α) %. Multivariate Cox models will also be conducted to explore the factors influencing QFS. All clinical and socio-demographic variables collected at baseline will be tested in univariate analysis. Variables with a *p*-value < 0.20 will be eligible for the multivariate model. This model will consider the collinearity of the eligible variables and will be constructed according to the Peduzzi rule of one variable for 10 events. The group will be forced into the multivariate model. Overall survival is defined by the interval between the date of randomization and the occurrence of death whatever the cause. It will be estimated according to the Kaplan-Meier method and described per randomized groups using the median and 95% confidence interval, and compared between the two groups using the Log-rank test. A univariate Cox model will be performed to estimate the Hazard Ratio coefficient and its 95% confidence interval.

### Qualitative study

The first part of the qualitative study focuses on patients’ experiences of care, aiming to better understand the specificity of patients’ perceptions of PC between outpatient consultation and day hospital. We will explored dimensions of organisation and process of care that contribute to satisfaction among patients received either PC models. This study is to take place in only one participating centre (Institute Curie), as heterogeneity across cancer centres is unlikely. Approximately 10 to 15 patients randomly selected from each intervention group will be interviewed 2 months after their inclusion by a research psychologist on their experiences and satisfaction with the palliative care provided. The recorded interviews will be transcribed and a qualitative analysis will be carried out using grounded theory constant comparison techniques [48, 49]. The semi-structured interviews will elicit patients’ care experiences with care providers and factors associated with PC, focusing on their perceptions of the medical and nursing care relationship: listening and support, the information and advice provided, the logistic aspects and continuity of care, the attention given to their loved ones and the impact of the care delivered. It will also explore their coping patterns and the role that PC could play in their psychological adjustment. Patients will be invited to talk about critical episodes or on the contrary particularly positive care experiences, and to provide any comments deemed useful to improve care.

The second part of the qualitative study focuses on PC health professionals in day hospital settings in order to understand organizational and professional aspect of PCDH work. To avoid a bias caused by specific features of one particular PC team, this survey will take place in two of the 5 participating cancer centres. Interviews will be conducted by two research sociologists, with PC physicians and nurses, and will concern the care of one or two patients randomly selected among patients seen the previous week, every week for 5 to 6 months. Research continues in cumulative mode until saturation is obtained, using grounded theory constant comparison techniques [48, 49]. Thus, an interactionist sociological survey will be undertaken concerning medical work in the PDCH (20). Data will be collected to reconstitute patient history, and questions will then focus on the description of the patients’ needs for supportive care, the PC discussions that took place, shared medical decisions, and the PC team’s feelings concerning the quality of care and relationships with the patients and their relatives.

### Medico-economic study

The study will be carried out from a societal point of view. A cost estimate will be made by collecting actual care consumption from the patients’ files for the last 3 months of life and assumptions will be made to estimate costs between inclusion in the study and the last 3 months of life.

Only costs that discriminate between the two strategies will be taken into account. Direct medical costs are costs directly attributable to the pathology and/or its treatment: care and medical and social expenditure.

These costs will include:

- Consultations carried out by community health (source of price data: fees established in General classification system for the professional activities, NGAP);

- Expensive medical and technical procedures such as MRI, or scanners (source of price data: fees established in the Common Classification of Medical Acts, CCAM);

- Hospitalization at home (source of price data: National average DRG related stay cost, ENCC 2016);

- The professionals’ interventions from the palliative care network or the mobile palliative care team (source of price data: bundled payment received by Hospital for public missions, MIGAC);

- Hospitalization in health care institutions, including rehabilitation units, acute medical care, etc. (source of price data: bundled payment received by Hospital for public missions, MIGAC);

- Care in day hospitalization (source of price data: National Cost Study 2016);

- Consultations in medical emergencies (source: of price data National average DRG related stay cost, ENCC 2016);

- Consumption of chemotherapy or other anti-cancer treatment (source of price data: Drug tariff database, BDM-IT);

- Medical transport expenses (source of price data: tariff established by the National Health insurance; the use of non-medicalized and non-healthy transport will be valued according to the rate per kilometer of transport and the distances travelled per kilometer).

A cost-effectiveness analysis will be conducted using the Global Survival effectiveness criteria.

The scores from the validated, specific HRQoL questionnaire EORTC QLQ-C30, will be converted into a score for the EuroQol 5 Dimensions questionnaire (EQ. 5D) [50] for inclusion in a cost-utility analysis. Another analysis will be carried out using a questionnaire that measures well-being according to the capability approach, the ICECAP-SCM [44]. This questionnaire has not yet been translated and validated in French. Therefore, this study will also contribute to the validation of this necessary tool for conducting medical and economic evaluations in the field of palliative care.

## Discussion

Several PC types of structure are usually described for integrated PC in oncology: inpatient consultation, outpatient clinics, palliative care units, community-based palliative care and hospice care (30). In a survey from 184 ESMO designated cancer centers of integrated PC there were present at 90, 89, 71 and 50% respectively [51]. In this survey 70% of patients with advanced cancer had a PC consultation before death, occurring 90 days before death for outpatients and 21 days for inpatients and 118 (78%) reported that routine symptom screening was offered in the oncology. Outpatient PC can facilitate timely referral but the optimal model of outpatient palliative care is not known. There are currently many variations for how PC is delivered in the outpatient setting, which can be described with some key characteristics: type of specialized PC staff (physician and/or nurse), team makeup (with or without psychologist, chaplain and social worker), place of in intervention (in oncology clinics or in stand-alone clinics), variously embedded joined PC and oncology staff during consultation, mixed PC and oncology meetings or other modalities of education [52, 53]. The effect of an embedded advanced practice PC nurse has been assessed only with non-randomized studies and small sample size, and the heterogeneity of the models of PC delivery made analysis difficult [54, 55]. In the same way, the effect of an embedded PC team with a physician and a nurse was assessed with a pre/post study design [56–58]. At this time, interdisciplinary specialist PC in stand-alone clinics remains the gold standard for ambulatory palliative care because this approach has the greatest impact on multiple patient and caregiver outcomes [52]. Regarding the particularly promising conditions for PC delivery in these structures, PCDH could be the optimal place for outpatient palliative care. One of the strengths of PCDH is the interdisciplinary PC approach aiming to provide symptoms relief, psychosocial support, education and shared decision making, responsibility and advance care planning. The physician, nurse, psychologist, social worker, chaplain, pharmacist, physiotherapist, occupational therapist, and other allied health professionals each contribute their unique expertise while working together in a cohesive manner to support the patient’s goals of care through impeccable assessments, coordinated communication, and multidimensional interventions. Not all members are required at all times, some may be needed more often than others, and some may form a closer relationship with the patient. Moreover, PCDH setting to deliver integrated PC promote concertation between oncologists, PC with or without patients and relatives, and care coordination with home health caregivers. Progress in cancer therapy over the past two decades, particularly in targeted therapies or immunotherapy, has focused on extending the life expectancy of metastatic patients with incurable metastatic disease rather than on the rates of cure [59]. As integrated PC is a gold standard of care, this has led to a considerable increase in the need for outpatient palliative care facilities in cancer centres [12]. In addition, hospitalization in oncology is proving more and more difficult in some places. Consequently, if the results of our trials confirm the major role of PCDH in outpatient PC delivering, this could lead to national guidelines to develop these structures in all comprehensive cancer centres.

To the best of our knowledge, this is the first randomized controlled trial to set out to compare PCDH with usual palliative care. This study will broaden perspectives in ambulatory palliative care and will promote PCDH as a new palliative care setting. Randomisation will give a strong weight to eventually positive results. Problems associated with conducting RCT in PC are well documented. Despite these difficulties, the improvement of evidence base of PC is essential and RCTs are a recognized method to provide these proofs [60, 61]. Another strength of this project is its design with a mixed-method: a randomised clinical trial testing the hypothesis of an impact of the PCDH on HRQoL and a qualitative study understanding PC organization and exploring factors that could promote or impede the efficacy of specific palliative care facilities in a hospital cancer unit. Indeed, mixed-methods research is becoming an important methodology to investigate complex health-related topics and gain a more complete understanding of systems in health [62]. In a context of the control of medical health expenditure, the biggest challenge is to find the right combination in order to improve quality of care and both control structural costs. The medical and economic study in our trial will contribute to the assessment of the effectiveness and economic consequences of PCDH compared to PC ambulatory consultations. Therefore, our study will broad the ambulatory palliative care perspectives and will promote the PCDH as a new PC outpatient setting.

## Data Availability

Not applicable.

## Abbreviations

ASCO: American society of clinical oncology
ECOG: East cooperative oncology group
EOL: End-of-life
EORTC QLQ-C30: European Organization for research and treatment of cancer quality of life questionnaire core30
EQ 5 D: EuroQol 5 dimensions
HADS: Hospital anxiety and depression scale
HRQOL: Health-related quality of life
ICECAP-SCM: Index of capability supportive care measure scale
Out-PATSAT7: Out-patient satisfaction 7
Out-PATSAT-C33: Out-patient satisfaction Core33
PC: Palliative care
PCDH: Palliative care day-hospital
PTPQ: Prognosis and treatment perception questionnaire
QFS: Quality of life survival deterioration free survival
RCT: Randomized controlled trial
SCNS-P&C-F: Supportive care needs survey for partners and caregivers French version
